# Peripheral primitive neuroectodermal tumor: a case report

**DOI:** 10.1186/s13256-022-03354-2

**Published:** 2022-03-31

**Authors:** Alije Keka-Sylaj, Atifete Ramosaj, Arbana Baloku, Leonore Zogaj, Flamur Mushica, Fisnik Kurshumliu

**Affiliations:** 1 Institute of Anatomy, Pediatric Clinic, Faculty of Medicine, University of Prishtina, Prishtina, 10000 Kosovo; 2 Pediatric Clinic, University Clinical Center of Kosovo, Prishtina, 10000 Kosovo; 3 Department of Radiology, Langenthal Hospital, St. Urbanstrasse 67, Langenthal, 4900 Basel, Switzerland; 4 Institute of Pathology Anatomy, Faculty of Medicine, University of Prishtina, Prishtina, 10000 Kosovo

**Keywords:** Primitive neuroectodermal tumor, Mediastinal tumor, Children, Musculoskeletal manifestations

## Abstract

**Background:**

Primitive neuroectodermal tumors are extremely rare and highly aggressive malignant small round cell tumors that arise from the primitive nerve cells of the nervous system or outside it. These tumors share similar histology, immunohistologic characteristics, and cytogenetics with Ewing’s sarcoma. Peripheral primitive neuroectodermal tumors of the chest wall are rare malignant tumors seen in children and young adults.

**Case presentation:**

We report a rare case of peripheral primitive neuroectodermal tumor in a 4-year-old Albanian girl with a mediastinal tumor and an unusual clinical presentation. She was initially treated for acute polyradiculoneuritis (Guillain–Barré syndrome) owing to pain, weakness in the lower limbs, and walking difficulty, as well as severe irritability. During the second week of treatment, the child began to experience dry cough, chest discomfort, and worsening dyspnea. Chest radiography, chest computed tomography, and contrast-enhanced computed tomography demonstrated a large mass in the right hemithorax that was derived from the posterior mediastinum with expansive growth in all directions and that shifted the mediastinal structures in the anterolateral left direction. Consequently, histopathology and immunohistochemical examination of the markers S-100, CD99, and Ki-67 showed that the tumor cells stained positively for S-100 and CD99. The proliferative index measured by Ki-67 was approximately 20%, which suggested primitive neuroectodermal tumor.

**Conclusions:**

Even though other diseases, including leukemia, lymphoma, and neuroblastoma, may be accompanied by musculoskeletal manifestations in children, other solid tumors, such as peripheral primitive neuroectodermal tumors, should be considered in the differential diagnosis in any child presenting with musculoskeletal symptoms.

## Introduction

Primitive neuroectodermal tumors (PNETs) are rare, highly aggressive, malignant small round cell tumors that arise from the primitive nerve cells of the nervous system; these are termed CNS PNETs or central PNETs (cPNETs), which occur in the form of medulloblastoma [1]. They can also occur outside the central nervous system (peripheral PNETs), including in the chest wall, paravertebral region, pelvis, and extremities; they are presumed to be of neural crest origin when they arise outside the central and sympathetic nervous systems [2]. Peripheral PNETs (pPNETs) typically occur in children or young adults, with a slight predilection in males, although they can occur at other ages [3]. Primary mediastinal malignant tumors are rare and represent only approximately 3% of all chest tumors [4]. Among these uncommon malignancies, peripheral primitive neuroectodermal tumors (pPNETs) are even more infrequent [4]. Here, we report a case of pPNET in a 4-year-old girl who presented with a posterior mediastinal tumor with an unusual clinical presentation and poor prognosis.

## Case presentation

A 4-year-old Albanian girl from a rural area was referred to the Department of Neurology of the University Clinical Centre of Kosovo because of abdominal pain, progressive weakness in the lower limbs, and walking difficulty. Prenatal and postnatal history: She was the fourth child in the family and was born as a result of a full-term pregnancy that was not well controlled. No history of drug use or exposure to radiation during pregnancy was reported. Delivery was completed at a regional hospital; she had a birth weight (BW) of 3500 g. Treatment with retinol and cholecalciferol (AD3) for prevention of vitamin D deficiency and vaccinations were irregular. She was breastfed for 1 year, and supplementary food was introduced at 5 months. Her growth and development were normal, and this was her first hospitalization. Family history: Her aunt died from breast cancer at 35 years of age.

Disease history: The first symptoms appeared 2 months before admission and consisted of profuse sweating of the head, neck, and upper part of the body, mostly at night, followed by progressive abdominal pain. Therefore, she sought medical attention and was treated with analgesic and antiparasitic therapy. Despite treatment, the abdominal pain persisted, became more progressive, and was followed by musculoskeletal manifestations in the lower limbs. In general, the physical examination at admission revealed a relatively good general condition; she weighed 15 kg and was conscious, alert and communicative. Moreover, a physical examination of the musculoskeletal system revealed no erythema or swelling, but a reduced range of motion in the lower limbs with no weight- bearing tolerance was observed. In addition, muscle tonus was decreased in the lower limbs, whereas it was within the normal range with intact sensory perception in the upper limbs. The abdomen was slightly distended, soft upon palpation, and not painful. Furthermore, the patient’s blood pressure was within the normal range for her age; likewise, clinical findings in other systems were within normal limits.

Initially, the results of routine blood tests showed a normal blood cell count; hemoglobin (Hgb) was 11.2 g/dl, and platelet count was 249,000/mm^3^. The erythrocyte sedimentation rate was 40 mm per hour, and C-reactive protein was negative [1.2 mg/l, normal range (NR) > 6 mg/l]. Furthermore, liver enzymes, coagulogram, urea and creatinine, and acid–base status of the blood were normal. However, her lactate dehydrogenase (LDH) level of 536 U/l at admission continued to increase to 3520 U/l (NR 160–450 U/l). In addition, a progressive decrease in total serum proteins from 6.0 to 2.9 g/dl (6.3–8.0 g/dl) was observed. Other investigations, including urinalysis and urine and blood culture, were negative; abdominal ultrasound was also unremarkable.

Owing to progressive weakness in the lower limbs and walking difficulties, this patient was initially suspected to have acute polyradiculoneuritis (Guillain–Barré syndrome), and thus, lumbar puncture was performed. Cytology and biochemical analysis of cerebrospinal fluid (CSF) revealed white cells 10 cells/mm (100% monocytes) and proteins 44.1 g/dl (NR 15–40 g/dl), while the CSF glucose level was normal compared with the plasma values. Moreover, electromyoneurography (EMNG) revealed a reduced amplitude and velocity of the M wave for the peroneus and tibial nerve on both sides.

**Fig. 1:**
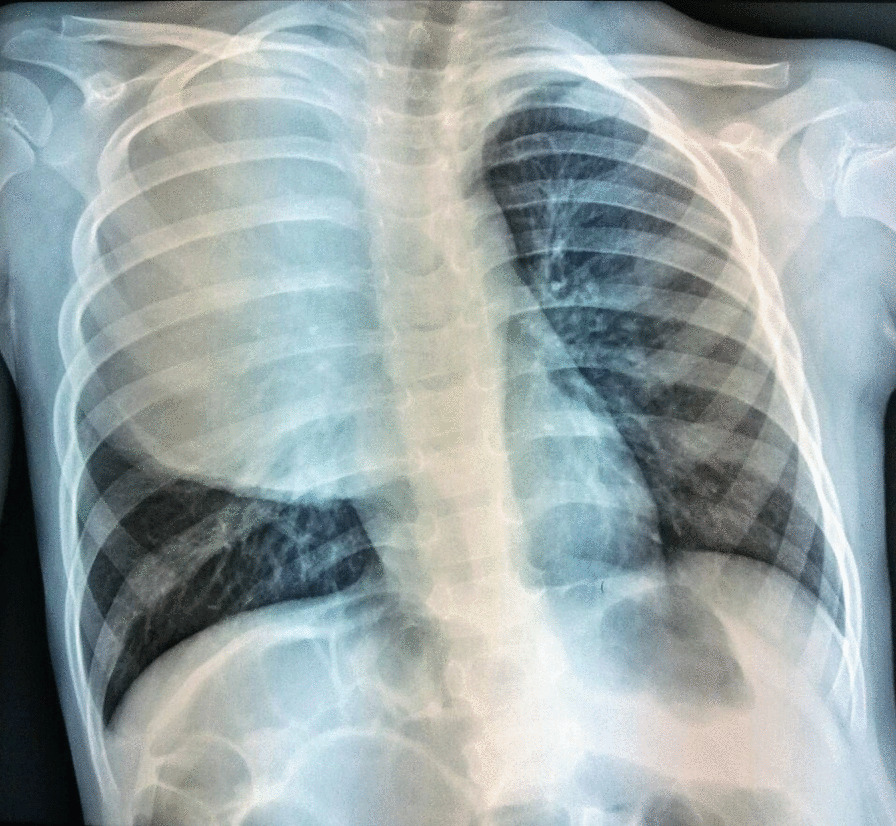
Frontal chest radiograph showing a large homogeneous mass occupying the entire right hemithorax, with a slight mediastinal shift to the left, which suggests a posterior mediastinal location

Therefore, she was suspected to have polyradiculoneuritis and was treated with corticosteroids and immunoglobulins. However, this medication did not improve her clinical condition. Instead, during the second week of treatment, the child began to experience dry cough, chest discomfort, and worsening dyspnea. Therefore, she was transferred to the intensive care unit. Chest radiography revealed a hyperdense homogeneous shadow occupying almost the entire right hemithorax with a slight mediastinal shift to the left, which suggested a massive right-sided mass, while no pleural effusion was noted (Fig. 1).

**Fig. 2:**
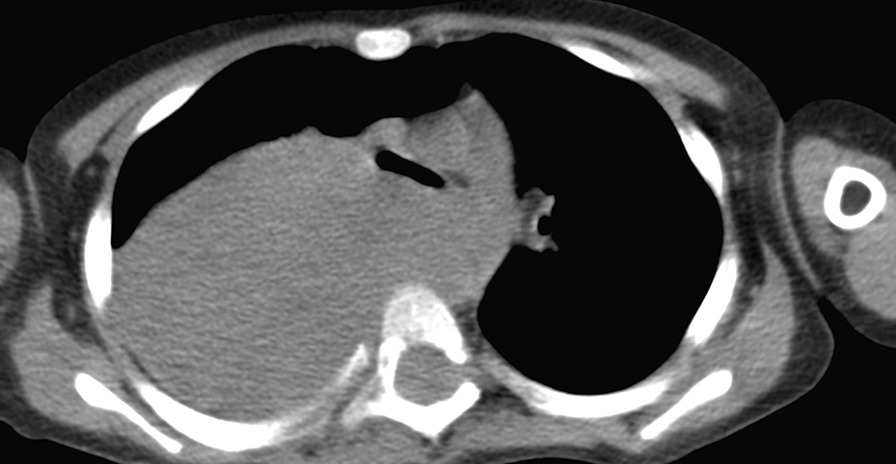
A plain axial chest computed tomography scan showing a large well-defined hypodense mass in the right hemithorax that was derived from the posterior mediastinum. Expansive growth was observed in all directions, which shifted the mediastinal structures in the anterolateral left direction

Subsequently, chest computed tomography (CT) was performed, which revealed a large mass of homogeneous density that may have originated in the posterior mediastinum; this mass occupied the entire right hemithorax. A left mediastinal displacement was described with compression of the principle bronchus and minimal pleural leakage (effusion) (Fig. 2).

Moreover, since tumors such as neuroblastoma were suspected, the levels of homovanillic acid (HVA) and vanillylmandelic acid (VMA) in urine were determined and were found to be negative. Furthermore, echocardiography revealed that the heart had shifted down and to the left without pericardial effusion. On the right side of the hemithorax, pleural effusion was noted around the homogeneous mass; however, when thoracentesis was performed, only 90 ml hemorrhagic fluid could be aspirated from the right infrascapular area after repeated attempts. A pleural fluid study yielded fluid with a reddish color, and cytological examination predominantly showed elements of peripheral blood, while the microbiological culture was negative.

**Fig. 3:**
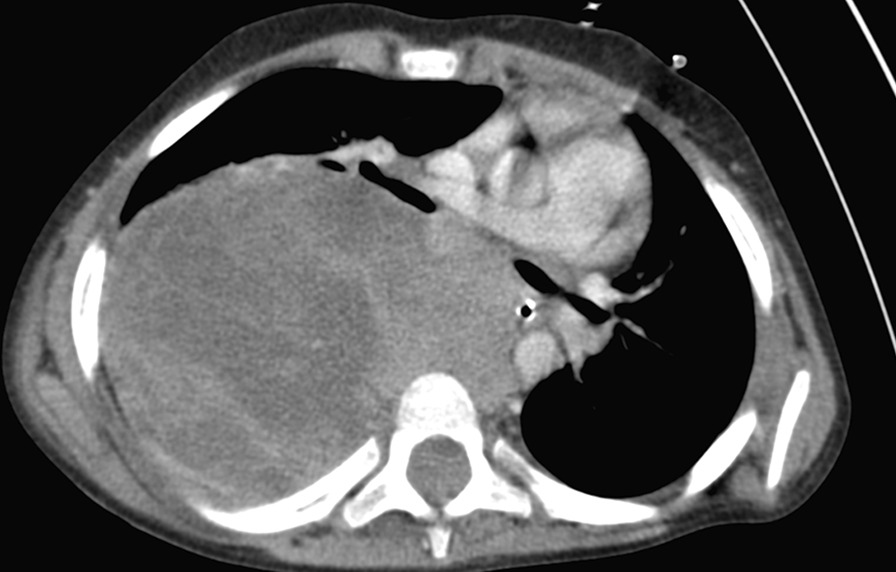
Axial contrast-enhanced computed tomography indicating the heterogeneous nature of the tumor with vital solid areas and central necrotizing areas. Blood vessels and aerogenic structures are shifted, but no signs of macroscopic invasion are observed. There are also no signs of bone destruction or a direct connection to the spinal canal. Imaging data are highly suggestive of peripheral primitive neuroectodermal tumors (pPNETs)

Furthermore, contrast CT of the thorax and abdomen revealed a large heterogeneously enhancing mass 95 × 75 × 70 mm in size in the right hemithorax, with central necrotic areas that likely originated from the outlet roots of spinal nerves of the spinal canal, which shifted the right lung and mediastinal organs. Minimal pleural effusion was observed in the phrenicocostal right sinus (hemorrhagic), with the drain located around the mass that was functional. Moreover, no adenopathy was detected in the mediastinum or axillary regions, and abdominal CT scans were unremarkable (Fig. 3).

**Fig. 4:**
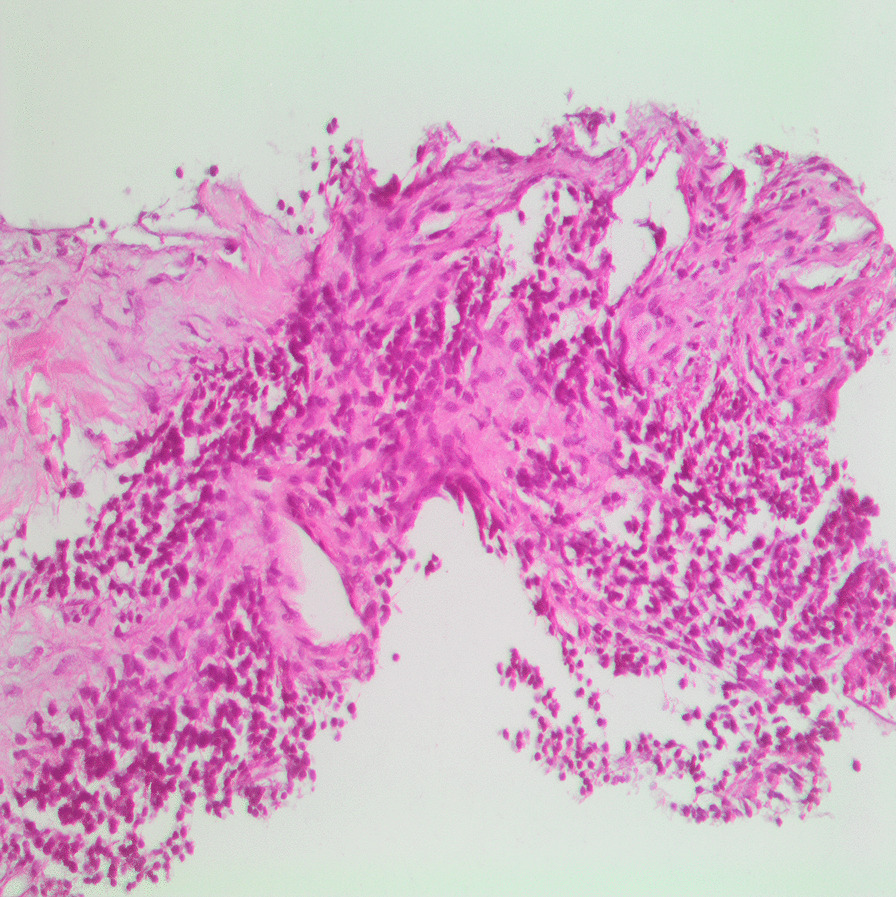
Histopathology and immunohistochemistry revealed small “round blue” tumor cells with hyperchromatic nuclei (hematoxylin and eosin, ×20 magnification)

**Fig. 5:**
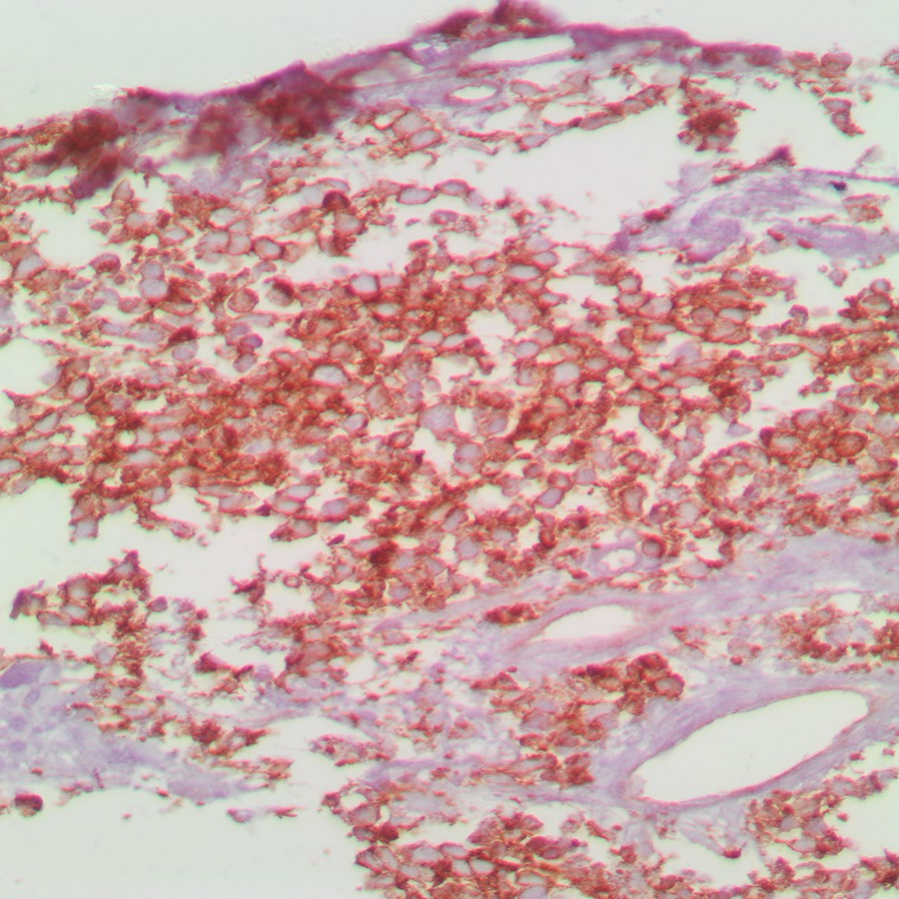
Immunopositivity for CD99 (immunoperoxidase, ×40 magnification)

**Fig. 6:**
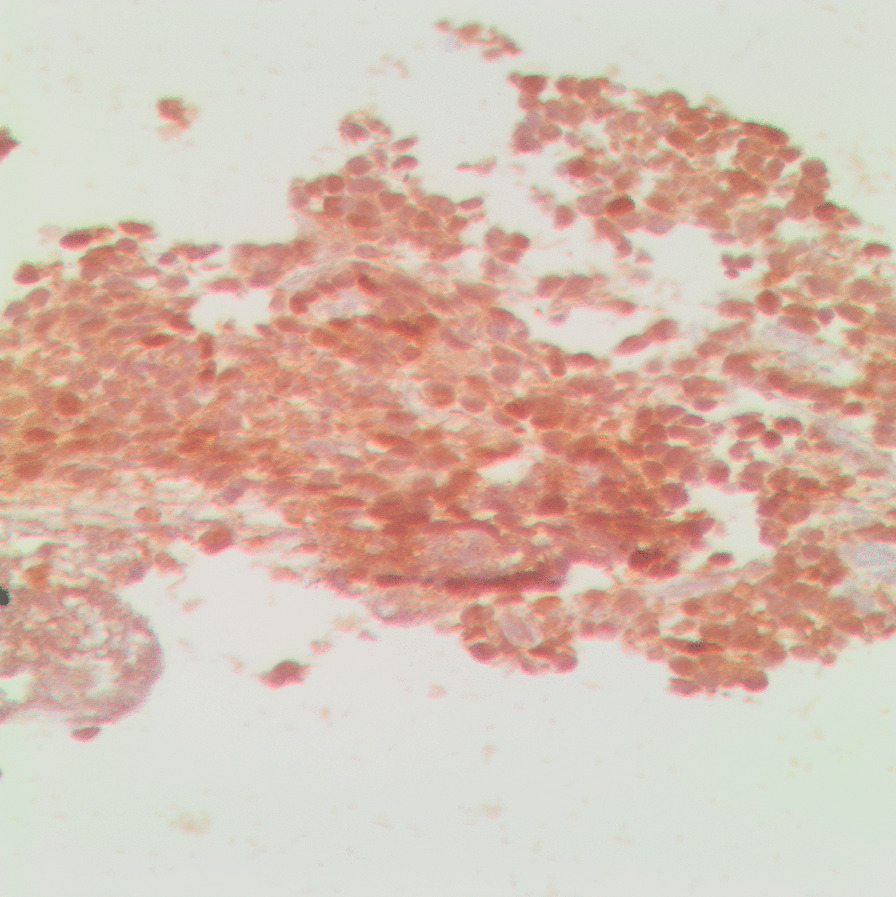
Immunopositivity for S-100 (immunoperoxidase, ×40 magnification)

**Fig. 7:**
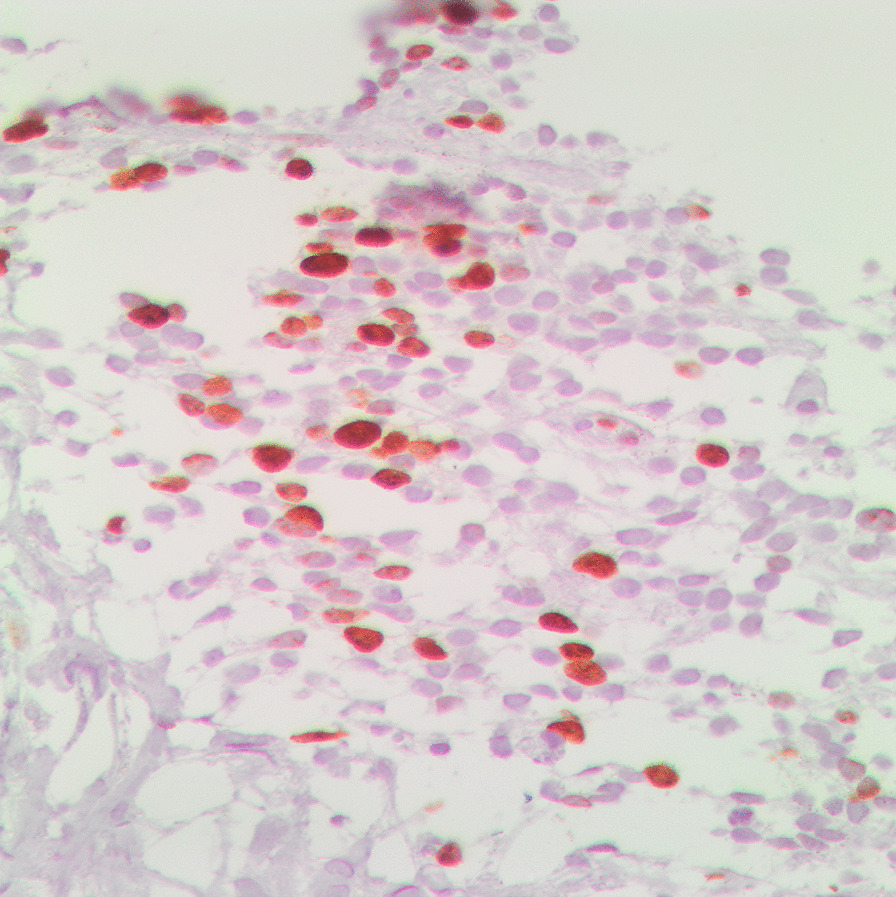
Proliferation index as measured by Ki-67 (immunoperoxidase, ×40 magnification)

For the histopathological diagnosis, during CT, a percutaneous needle biopsy of the right mass was performed. Consequently, the histopathology of the examined tissue cylinders identified tumor tissue composed of poorly differentiated malignant small round cells with dark staining, cells with round nuclei (“small round blue cells”), and a confluence of necrotic foci, which suggests the tumor type of blastoma (Fig. 4). Conclusively, immunohistochemical (IHC) examination of the markers CD99 (Fig. 5), S-100 (Fig. 6), and Ki-67 (Fig. 7) showed that the tumor cells stained positively for S-100 and CD99. Specifically, the proliferative index measured by Ki-67 was approximately 20%, which suggests primitive PNET.

## Discussion

PNET is a malignant neoplasm included in the Ewing’s sarcoma (ES) family of tumors owing to its biological similarities with osseous and extraosseous tumors arising from germinal matrix cells of the primitive neural tube [4]. Peripheral PNETs have primarily been observed in children in different organ systems, including bone [5, 6], mandible [7], the maxillofacial area [8], eye [9], parathyroid gland [10], thymus [11], thorax [1, 12], lung [13], urogenital tract [14–17], adrenal gland [18], pancreas [19], chest wall [20], and subcutaneous abdominal wall [21].

Additionally, only a few case reports have described peripheral PNETs in the posterior mediastinum in adults [22–25], as these tumors are commonly seen originating from the chest wall (“Askin tumor”), according to the first description of malignant small cell tumors of the thoracopulmonary region in 20 pediatric patients by Askin *et al*. [26]. To the best of our knowledge, this is an extremely rare case of pPNET in the posterior mediastinum in a child of this age with an atypical clinical presentation.

In general, the clinical presentation of this disease is not pathognomonic and depends on the affected site, the degree of tumor invasion and the involved structures [27]. Moreover, pain and tenderness often lead to complaints in cases of bone and soft tissue tumors alike, together with paresthesias, weakness, or loss of function, primarily when nerves are involved [28]. Likewise, other signs and symptoms at presentation may include pain, cough, and respiratory distress. Fever may be present, which is suggestive of an infection that is most commonly acute osteomyelitis [29].

In our case, the clinical manifestations were initially atypical with profuse sweating, mostly at night, and abdominal pain, which progressed during the following 2 months with pain onset prior to weakness in both lower limbs and walking difficulty progressing up to an inability to walk. Moreover, cough, chest discomfort, and worsening dyspnea due to chest wall extension of the mediastinal lesion were observed almost 3 months after the onset of the first signs and symptoms, as the tumor had grown to a large size and compromised the structures of the surrounding organs. Although the histogenesis of this tumor remained uncertain, based on CT scans, it is suspected that the tumor arose from the outlet roots of spinal nerves, which may explain the musculoskeletal manifestations.

For diagnostic purposes, imaging studies should include magnetic resonance imaging (MRI) and CT. However, radiologic findings are nonspecific for the differentiation of PNETs from other types of bone and soft tissue tumors. Nevertheless, in general, their typical appearance resembles large noncalcified, soft tissue masses containing cystic or necrotic areas with heterogeneous contrast enhancement [30].

Importantly, a definitive diagnosis requires histopathology and immunohistochemistry. Generally, the classic histological pattern of ES/PNET consists of solid sheets of small uniform “primitive” cells, which have round nuclei and scant cytoplasm and lack significant differentiation [31]. Furthermore, these tumors consist of small round cells with rounded nuclei, fine chromatin, and eosinophilic cytoplasm. Moreover, undifferentiated neuroectodermal tumors are indicative of ES, while PNET is reserved for differentiated tumors [32]. The presence of mitotic figures, necrosis, endothelial hyperplasia, and Homer–Wright rosettes favors a diagnosis of PNET instead of ES [32].

In particular, by immunohistochemical analysis, PNETs are positive for the surface antigens CD99, 12E7, E2, 013, and HBA71, which are all products of the *MIC*2 gene; in this regard, staining is very helpful for diagnosing these tumors. Likewise, immunoreactivity for synaptophysin, NSE, PGP9.5, vimentin, S100, and neurofilament, which indicates neuroectodermal differentiation, may also be observed and supports the diagnosis of pPNET [32]. Tumor cells of thoracopulmonary PNETs are CD99-positive but cytokeratin-negative. Notably, CD99 positivity has 95% sensitivity for PNET [33].

The differential diagnosis for a posterior mediastinal mass in children and adolescents should include sympathetic ganglion tumors (neuroblastoma, ganglioneuroblastoma, ganglioneuroma) and nerve sheath tumors (schwannoma, neurofibroma, malignant peripheral nerve sheath tumors). Likewise, rhabdomyosarcoma, non-Hodgkin lymphoma, and Langerhans cell histiocytosis should also be considered in the differential diagnosis [8]. Concerning the treatment, due to the aggressive behavior of the neoplasm and its great potential to metastasize, treatment should be multimodal and involve radical surgical resection, radiotherapy, and chemotherapy [34].

Unfortunately, the clinical status of our patient worsened during her hospital stay, and she developed respiratory distress and died before starting the specific treatment.

Generally, pPNETs are aggressive, and significant prognostic factors include tumor site, tumor volume, and presence of metastasis [35]. Some factors have been associated with poor outcomes, including tumor volume > 100 cm^3^, axial location, increased LDH levels, low serum albumin levels, metastasis, older age, and neural differentiation [27].

## Conclusion

Our case is a rare example of a childhood malignancy with onset of atypical manifestations and progression of musculoskeletal symptoms. Even though other diseases, including leukemia, lymphoma, and neuroblastoma, can be accompanied by musculoskeletal manifestations in children, other solid tumors, such as pPNETs, should be considered in the differential diagnosis in any child presenting with musculoskeletal symptoms.

## Data Availability

All of the data and materials will be available from the corresponding author upon request.

## Abbreviations

AD3: Retinol and cholecalciferol
BW: Birth weight
CNS PNET: Central nervous system primitive neuroectodermal tumor
CSF: Cerebrospinal fluid
CT: Computed tomography
EMNG: Electromyoneurography
ES: Ewing’s sarcoma
Hgb: Hemoglobin
IHC: Immunohistochemical
LDH: Lactate dehydrogenase
MRI: Magnetic resonance imaging
NR: Normal range
PNET: Primitive neuroectodermal tumor
